# Multifunctional all-dielectric quarter-wave plate metasurfaces for generating focused vector beams of Bell-like states

**DOI:** 10.1515/nanoph-2023-0923

**Published:** 2024-03-06

**Authors:** Guosen Cui, Manna Gu, Chen Cheng, Ziheng Zhang, Yuxiang Zhou, Qingrui Dong, Song Gao, Duk-Yong Choi, Chuanfu Cheng, Chunxiang Liu

**Affiliations:** School of Physics and Electronics, 47856 Shandong Normal University, Jinan, 250014, China; School of Information Science and Engineering and Shandong Provincial Key Laboratory of Network Based Intelligent Computing, 12413 University of Jinan, Jinan, Shandong 250022, China; Laser Physics Centre, Research School of Physics and Engineering, 2219 Australian National University, Canberra, ACT 2601, Australia

**Keywords:** vector beams, metasurfaces, quarter-wave plates, light field manipulation

## Abstract

The generation of vector beams using metasurfaces is crucial for the manipulation of light fields and has significant application potential, ranging from classical physics to quantum science. This paper introduces a novel dielectric metasurface composed of quarter-wave plate (QWP) meta-atoms, known as a QWP metasurface, designed to generate focused vector beams (VBs) of Bell-like states under right circularly polarized illumination. The propagation phase imparted on both the co- and cross-polarized components of the output field constructs hyperbolic and helical phase profiles with topological charge *l*_*p*_, whereas the Pancharatnam–Berry (PB) phase acts only on the cross-polarized component to construct another helical phase profile with topological charge *l*_*g*_. Thus, the co- and cross-polarized components form two orthogonal vector vortex (VV) modes with topological charges *l*_*p*_ and *l*_*p*_ + *l*_*g*_, respectively. When the parameter conditions are satisfied by matching the incident polarization chirality *σ* and topological charges *l*_*p*_ and *l*_*g*_, the focused VBs of Bell-like states are generated by simultaneously manipulating the two VV modes, in contrast to existing QWP metasurfaces. The polarization states of the generated VBs are manipulated using the initial orientation angle *θ*_0_ of the meta-atom. Overall, this research provides an innovative strategy for metasurface design, enhancing the functionality of metasurface devices for a broader range of application scenarios.

## 1 Introduction

With spatially variant polarization distributions, vector beams (VBs) represent an important type of structured light [1], with wide ranging applications in both classical and quantum fields owing to their unique properties. Classic applications include laser processing [2], optical encryption [3], and clinical and medical detection [4]. In classical communications, the spatial modes of the VBs serve as information channels carrying independent data streams. The use of polarization multiplexing [5] and mode-division multiplexing [6] helps enhance the transmission data rates and information capacity of free-space optical communication, achieving low mode crosstalk and bit error rates. VBs, with their non-separable coupling of spatial and polarization degrees of freedom, similar to the local entanglement in a bipartite system [7], [8], are also commonly referred to as classically entangled light and have been widely used in quantum walk [9], quantum error correction [10], and quantum state tomography [11]. Additionally, VBs have been exploited as a novel resource for quantum information protocols to encode rotationally invariant qubits in long-distance alignment-free quantum communications [12], [13]. In particular, the four bases of first-order spin–orbit modes with maximal non-separability, that is, |TM⟩_1_, |TE⟩_1_, |HE^e^⟩_1_, and |HE^o^⟩_1_, have attracted considerable interest because of their special focusing properties. These entities are known as VBs of Bell-like states owing to their similarity to Bell states in quantum mechanics [14], [15]. In recent decades, various methods for VB generation have been established using different optical devices, such as Sagnac [16], Mach–Zehnder [17], or Michelson interferometers [18] containing q-plates [19], waveplates [20], and Daman gratings [21]. However, the bulky footprints of these elements restrict the system miniaturization and integration.

Metasurfaces consisting of subwavelength metallic/dielectric structures have been demonstrated to efficiently manipulate light fields in terms of amplitude, phase, and polarization, providing a promising platform for controlling complex structured light at subwavelength scales. As a novel compact device, metasurfaces have been widely used in various fields, such as high-resolution holography [22], [23], broadband achromatic lenses [24], dispersion-engineering [25], and information encoding [26], [27]. Among these fascinating applications, metasurfaces have emerged as a powerful tool for generating nanoscale VBs in the subwavelength dimension owing to strong spin–orbit interactions of light with the constituent meta-atoms. Earlier designs focused on generating radially and azimuthally polarized VBs by directly manipulating the local polarization and phase of the output light fields of the nanostructures [28], [29]. Subsequently, based on the concept of higher-order Poincaré (HOP) sphere, Yue et al. [30] introduced a metasurface design strategy to generate VBs by superposing two orthogonal circularly polarized (CP) vortices. This method has been extensively applied in metasurface design to generate versatile VBs, including multi-channel VBs [31], [32], [33], VB arrays [34], and perfect HOP beams [35]. However, these investigations focused on the generation of unfocused VBs without a hyperbolic lens phase profile. Notably, focused VBs have attracted significant interest owing to their unique focusing properties. A representative example is the radially polarized VB, which can be highly focused on a sharp spot beyond the diffraction limit with a strong longitudinal component [36]. Such focused VBs have potential applications in particle trapping [37], high-resolution metrology [38], and lithography [36]. In addition, focused VBs have contributed to recent discoveries of exotic optical phenomena near the foci, such as Möbius strips [39], and topological links and knots [40].

Unlike conventional optical waveplates with bulky sizes and limited functionalities, anisotropic meta-atoms can promote full-range form birefringence regulation at any desired wavelength through strong light–matter interactions. In the last decade, various metasurfaces consisting of waveplate meta-atoms with excellent performances and diverse functionalities have been proposed [41]. Nevertheless, most multifunctional waveplate meta-atoms have been centered on half-wave plates (HWP) [38], [42], [43], in which only the cross-polarized component of the output field can be manipulated, and the residual co-polarized component may interfere with the output fields as a noisy signal. In these investigations, the spin-decoupled HWP metasurfaces, which are capable of manipulating independently the two orthogonal CP components, have been extended to wide applications with multiple functionalities [44]. Specifically, the spin-decoupled HWP metasurfaces combining the propagation with Pancharatnam–Berry (PB) phases decouple the spin-locking originated from the spin-symmetric PB phase, achieving high efficiency and low-crosstalk [44], [45]. For VBs to be generated by such metasurfaces, the incident light needs to be linearly polarized (LP) to contain the two CP beams. The two corresponding cross-polarized components form the two orthogonal VV modes with different topological charges, and their superposition generates the hybrid-order Poincaré beams [46] rather than HOP beam [47]. In contrast, the QWP-meta-atom can simultaneously control both the co- and cross-polarized components, increasing the number of functional channels and information capacity, by leveraging the wavefield powers of the two orthogonal components. Initial applications of QWP-meta-atoms involved the design of single-layered metallic metasurface devices [48], [49], [50], later extended to gap-surface plasmon metasurfaces [51], [52], [53], to enhance the conversion efficiency through a reflectance configuration instead of transmission. Subsequently, all-dielectric metasurfaces consisting of anisotropic meta-atoms with high refractive indices and low losses garnered significant research attention. Recently, various dielectric metasurfaces consisting of QWP-meta-atoms with high conversion efficiencies have been developed for specific functionalities, such as polarization conversion [54], beam steering [55], meta-holography [56], [57], metalenses [58], optical encryption [59], [60], and vortex-beam generation [55], [58], [61], [62]. Some researchers have also attempted to use plasmonic QWP-metasurfaces to generate VBs under CP illumination [30], [63], [64]. However, in these applications, only the cross-polarized component was controlled by the PB phase to form a vortex beam, whereas the co-polarized component remained a bright spot without phase modulation. Correspondingly, hybrid-order VBs were generated by the interference superposition of the vortex and bright spots.

In particular, QWP-metasurfaces can not only manipulate the cross-polarized component using a combination of the propagation and PB phases to construct a helical phase profile, similar to HWP-metasurfaces, but can also control the co-polarized component by utilizing the propagation phase to set another helical phase profile. For the VBs to be generated by the QWP metasurfaces, merely one CP beam is used as the incident light, and the co-polarized and cross-polarized components may act as the two orthogonal VV modes to be independently manipulated. The two VV modes can carry the topological charges with equal values and opposite signs through properly matching the topological charges of the propagation and PB phase profiles, realizing the generation of the HOP beams. However, limited efforts have been made to simultaneously control both co- and cross-polarized components to generate focused VBs with QWP all-dielectric metasurface.

Therefore, this paper proposes a novel dielectric metasurface incorporating QWP-meta-atoms to generate focused VBs of Bell-like states, i.e., |TM⟩_1_, |TE⟩_1_, |HE^e^⟩_1_, and |HE^o^⟩_1_, under right circular polarization (RCP) light illumination. The proposed QWP-metasurface is different from existing frameworks that manipulated only the cross-polarized component, mostly through the PB phase, to realize certain simple functions, such as vortex generation, polarization conversion, and beam steering. In the proposed metasurface design, amorphous silicon (a-Si: H) meta-atoms are uniformly arranged on equally spaced concentric rings. The propagation phase imparted to both the co- and cross-polarized components is leveraged to construct hyperbolic and helical phase profiles with topological charge *l*_*p*_, and the PB phase imparted to only the cross-polarized component is used to yield a helical phase profile with topological charge *l*_*g*_. When the parameter condition *l*_*g*_ = 2*l*_*p*_ is satisfied by properly matching the incident polarization chirality *σ* and topological charges *l*_*p*_ and *l*_*g*_, the desired focused VBs can be generated by simultaneously manipulating the co- and cross-polarized components. Under RCP illumination with *σ* = 1, the co- and cross-polarized components form two orthogonal vector vortex (VV) modes with topological charges of equal absolute values but opposite signs. Specially, when *l*_*p*_ = 1, VV modes |*L*, −1⟩ and |*R*, +1⟩ are created, and their superposition generates a radially polarized VB. In contrast, when *l*_*p*_ = 1, the two VV modes are |*L*, +1⟩ and |*R*, −1⟩, the superposition of which produces a *π*-radially polarized VB. Thus, a complete set of focused VBs of Bell-like states can be obtained by setting the initial orientation angle *θ*_0_ of the meta-atoms. Additionally, when the light illuminating the same metasurface sample is changed to left circular polarization (LCP), the two orthogonal VV modes carry different topological charges owing to the chirality dependence of the PB phase, and their superposition generates focused hybrid-order VBs.

In this study, we first performed a theoretical analysis of the focused VBs based on Richards–Wolf vector diffraction theory (Section 2). Next, four all-dielectric QWP-metasurfaces were designed and fabricated based on appropriate parameter conditions for simultaneously manipulating two orthogonal VV modes. Finally, numerical simulations (Section 3) and experimental measurements (Section 4) were performed to demonstrate the flexibility and practicability of the designed multifunctional metasurface-integrated metalens focusing, polarization conversion, and VBs generation. Overall, this research yields a novel and effective method for designing multifunctional compact devices, which holds significant implications for the realization of metasurfaces with phase and polarization manipulation capabilities.

## 2 Theoretical analysis and metasurface design

**Figure 1:**
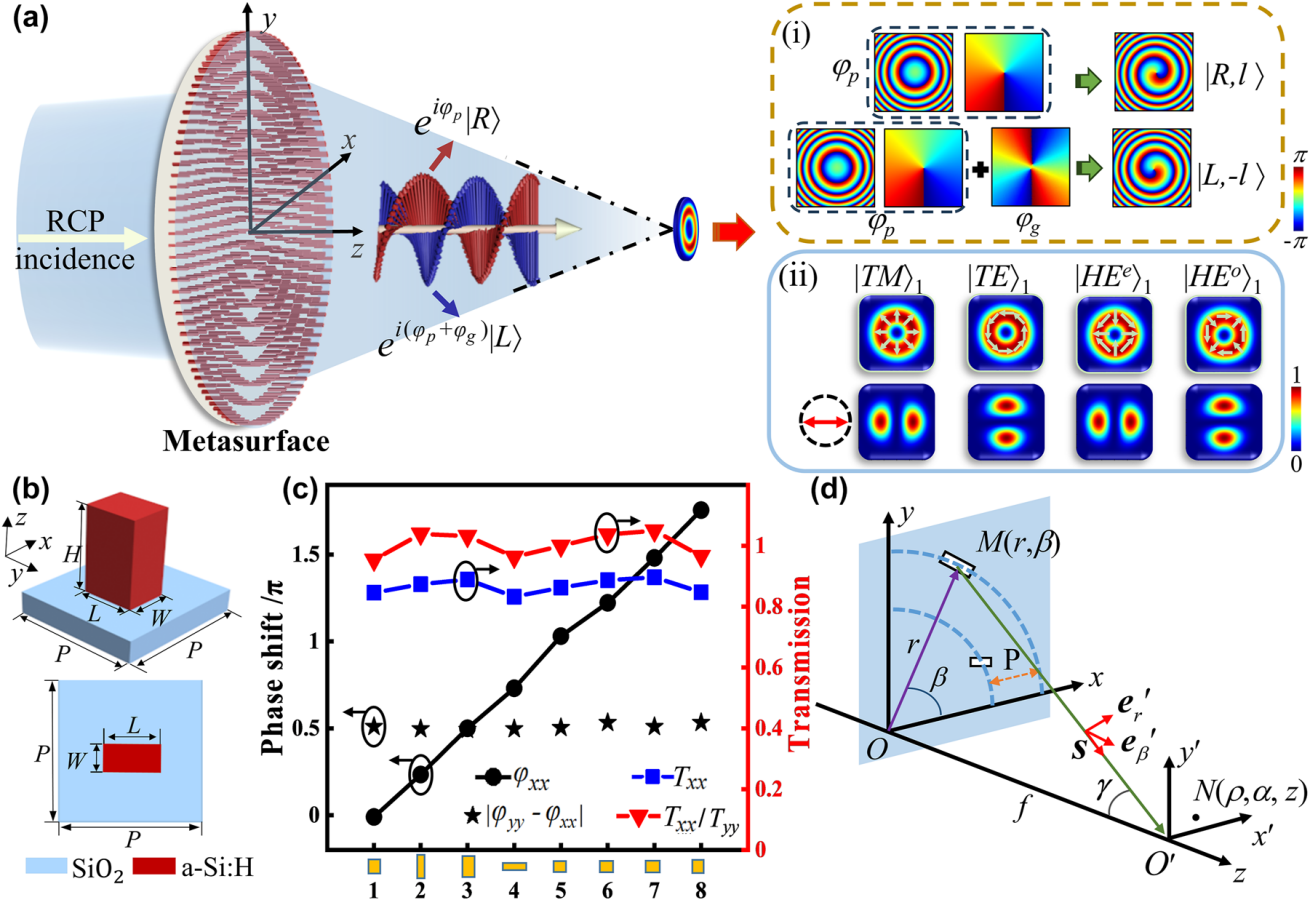
Demonstration of principle and method for metasurface design. (a) Schematic of an all-dielectric metasurface for generating focused VBs of Bell-like states. (b) Side and top views of a meta-atom. (c) Phase shift *φ*_*xx*_(*x*,*y*), phase retardation |*φ*_*yy*_−*φ*_*xx*_|, transmission amplitudes *T*_*xx*_, and transmission ratio *T*_*xx*_/*T*_*yy*_. (d) Illustrative geometry for the theoretical analysis of the focused VBs generated by the metasurface.

Figure 1(a) schematically illustrates the proposed metasurfaces consisting of QWP-meta-atoms for generating focused VBs of Bell-like states under RCP (with CP chirality *σ* = 1) illumination. The illustration focuses on the generation of |TM⟩_1_ as an example, and the phase profiles for the co- and cross-polarized components are intuitively outlined. Panel (i) in Figure 1(a) show the phase profile of the metasurface design. The propagation phase *φ*_*p*_(*x*,*y*) constructs a helical phase profile with azimuthally varying topological charge *l*_*p*_ and a radially varying hyperbolic phase, simultaneously controlling both the co- and cross-polarized components. The PB phase *φ*_*g*_(*x*,*y*) imparts another helical phase with topological charge *l*_*g*_, which acts only on the cross-polarized component. Consequently, the co- and cross-polarized components transform to two orthogonal CP vortex beams carrying topological charges of *l*_*p*_ and *l*_*p*_ + *l*_*g*_, respectively. When the parameter condition *l*_*g*_ = 2*l*_*p*_ is satisfied, the co- and cross-polarized components form two vortex eigenstates with topological charges of the same absolute value but opposite signs under RCP illumination, and VBs of Bell-like states are generated on the focal plane. Panel (ii) in Figure 1(a) schematically shows the intensity patterns of the total field and horizontal component of the focused VBs, with white arrows overlaying the doughnuts representing the polarization states. When LCP light is used to illuminate this metasurface, the PB phase and corresponding *l*_*g*_ are inverted, and the vortex topological charges of the co- and cross-polarized components are *l*_*p*_ and 3*l*_*p*_, respectively, resulting in hybrid-order VBs through the superposition of different VV modes. Figure 1(b) illustrates a rectangular dielectric meta-atom, consisting of an a-Si nanopillar on a silicon dioxide (SiO_2_) substrate of length *L*, width *W*, and height *H*. In the metasurface design, these meta-atoms are arranged on circular rings with an increment in the radii of two adjacent rings, that is, lattice period *P*. The integer number of meta-atoms on the same ring is calculated by dividing the perimeter by *P*. Figure 1(c) shows the finite-difference time-domain (FDTD) data curves of eight selected meta-atoms, that is, the propagation phase shift *φ*_*xx*_(*x*,*y*), phase retardation |*φ*_*xx*_(*x*,*y*)− *φ*_*yy*_(*x*,*y*)|, transmission amplitude *T*_*xx*_, and transmission ratio *T*_*xx*_/*T*_*yy*_. Figure 1(d) shows the geometry and coordinate relationships essential for the theoretical analysis of the focused VBs generated by the metasurface.

As a nanostructure with two perpendicularly symmetric axes, a meta-atom of rectangular nanopillar excites two wavefield modes polarizing along one axis or the other under the illumination of linear polarizations along the corresponding axis. The wavefields of the two modes experience different phase delays, resulting in the form birefringence. For a rectangular nanopillar with an arbitrary orientation, the interaction of light with a nanopillar meta-atom can be described by a Jones matrix:

(1) $$J\left(x,y\right)=R\left(-\theta \right)\left[\begin{array}{cc} T_{xx}{\text{e}}^{\text{i}\varphi_{xx}\left(x,y\right)} & 0\\ 0 & T_{yy}{\text{e}}^{\text{i}\varphi_{yy}\left(x,y\right)} \end{array}\right]R\left(\theta \right)$$

where *R*(*θ*) denotes the rotation matrix, *θ* is the orientation angle of the meta-atom relative to the *x*-axis, and the transmission amplitudes *T*_*xx*_ and *T*_*yy*_ are regarded as a constant value for the selected meta-atoms. When a meta-atom is illuminated with light of LCP |*L*⟩ and RCP |*R*⟩, the transmitted fields can be expressed as:

(2) $$J\left(x,y\right)\left|L\right\rangle =\exp\left[i\varphi_{p}\left(x,y\right)\right]\left|L\right\rangle +\exp\left[i\varphi_{1}\left(x,y\right)\right]\left|R\right\rangle$$

(3) $$J\left(x,y\right)\left|R\right\rangle =\exp\left[i\varphi_{p}\left(x,y\right)\right]\left|R\right\rangle +\exp\left[i\varphi_{2}\left(x,y\right)\right]\left|L\right\rangle$$

where *φ*_*p*_(*x*,*y*) = *φ*_*xx*_(*x*,*y*) is the propagation phase shift imparted on both co- and cross-polarized components; and *φ*_1_(*x*,*y*) and *φ*_2_(*x*,*y*) are the phases imparted on the cross-polarized components, consisting of the propagation and PB phases, respectively, depending on the chirality of the incident CP. From Eqs. (2) and (3), we can establish the Jones matrix of a meta-atom as follows:

(4) $$J\left(x,y\right)=\frac{1}{2}\left[\begin{array}{cc} {\text{e}}^{\text{i}\varphi_{1}\left(x,y\right)}+{\text{e}}^{\text{i}\varphi_{2}\left(x,y\right)}+2{\text{e}}^{\text{i}\varphi_{p}\left(x,y\right)} & i{\text{e}}^{\text{i}\varphi_{1}\left(x,y\right)}-i{\text{e}}^{\text{i}\varphi_{2}\left(x,y\right)}\\ i{\text{e}}^{\text{i}\varphi_{1}\left(x,y\right)}-i{\text{e}}^{\text{i}\varphi_{2}\left(x,y\right)} & -{\text{e}}^{\text{i}\varphi_{1}\left(x,y\right)}-{\text{e}}^{\text{i}\varphi_{2}\left(x,y\right)}+2{\text{e}}^{\text{i}\varphi_{p}\left(x,y\right)} \end{array}\right]$$

Because QWP nanopillars are used as the meta-atoms in our metasurface design, according to Eqs. (1) and (4), the propagation phase shift *φ*_*xx*_(*x*,*y*) and *φ*_*yy*_(*x*,*y*) along the two symmetry axes of the rectangular meta-atom, as well as the rotation angle *θ* of the fast axis relative to the coordinate axis, can be expressed as follows:

(5) $$\varphi_{xx}\left(x,y\right)=\left[\varphi_{1}\left(x,y\right)+\varphi_{2}\left(x,y\right)\right]/2+\pi /2$$

(6) $$\varphi_{yy}\left(x,y\right)=\left[\varphi_{1}\left(x,y\right)+\varphi_{2}\left(x,y\right)\right]/2+\pi$$

(7) $$\theta \left(x,y\right)=\left[\varphi_{1}\left(x,y\right)-\varphi_{2}\left(x,y\right)\right]/4$$

Notably, in addition to controlling the cross-polarized component, similar to commonly used metasurfaces consisting of HWP meta-atoms, QWP-metasurfaces can also manipulate the co-polarized component through the propagation phase. This property offers a novel mechanism for generating VBs with appropriate metasurface designs under CP light illumination. The propagation phase *φ*_*p*_(*x*,*y*) is used to construct the hyperbolic focusing and helical profiles, as follows:

(8) $$\varphi_{p}\left(x,y\right)=-\frac{2\pi }{\lambda }\left(\sqrt{r^{2}+f^{2}}-f\right)+l_{p}\cdot \beta$$

where *λ* is the wavelength, *f* is the focal length, *l*_*p*_ is the topological charge, and (*r*, *β*) represent the polar coordinates of the meta-atom at point *M* on the metasurface. Using the propagation phase specified by Eq. (8), both the co- and cross-polarized components simultaneously converge to the focus, and vortices with the same topological charge *l*_*p*_ are achieved. In addition, the PB phase *φ*_*g*_(*x*,*y*) is imparted only to the cross-polarized components to yield a helical profile with topological charge *l*_*g*_ = 2*σm*:

(9) $$\varphi_{g}\left(x,y\right)=2\sigma \theta =2\sigma \left(\theta_{0}+m\beta \right)$$

where *θ*_0_ is the initial orientation angle, *m* is the rotation order of the meta-atoms, and *σ* is the chirality factor of CP light. The propagation and PB phases in Eqs. (8) and (9) satisfy Eqs. (5)–(7). Therefore, based on the combination of the propagation and PB phases, the co- and cross-polarized components form two orthogonal CP vortices carrying topological charges of *l*_*p*_ and *l*_*p*_ + *l*_*g*_, respectively. Correspondingly, the generation of focused VBs on the focal plane can be realized by simultaneously manipulating the two VV modes. When the CP light of ***u*** ^*σ*^ illuminates a meta-atom with the Jones matrix defined in Eq. (1), the transmitted field can be obtained as:

(10) $$\begin{array}{ll} E^{\sigma }\left(r,\beta \right) & =J\left(x,y\right)\cdot u^{\sigma }\\ & =T_{xx}\left[{\text{e}}^{\text{i}\left(\varphi_{p}+\frac{\pi }{4}\right)}u^{\sigma }+{\text{e}}^{\text{i}\left(\varphi_{p}-\frac{\pi }{4}\right)}{\text{e}}^{\text{i}\varphi_{g}}u^{-\sigma }\right]/2 \end{array}$$

where $u^{\sigma }={\left[\begin{array}{cc} 1 & \sigma i \end{array}\right]}^{T}/\sqrt{2}$ . The light field near the focus can be considered the discrete sum of the wavelet fields diffracted from the meta-atoms. Based on the Richards–Wolf vector diffraction theory [65], the wave field near the focus *O*′ can be written as:

(11) $$E^{\sigma }\left(\rho ,\alpha ,z\right)=-\frac{ikf}{2\pi }\iint_{\Omega }\sqrt{\cos\gamma }E^{\sigma }\left(\gamma ,\beta \right){\text{e}}^{\text{i}k\left(s\cdot \rho \right)}\text{d}\Omega$$

where *k* denotes the wave vector, Ω denotes the solid angle, and dΩ = sin*γ*d*β*d*γ*. As shown in Figure 1(d), ***s*** is a unit vector along the propagation direction of the converging transmitted beam, and ***e***_*r*_′ and ***e***_*β*_′ are two orthogonal unit vectors located in the plane perpendicular to the propagation direction. For the observation point *N* near the focus, ***s*** ⋅ ***ρ*** = *z*cos*γ* + *ρ*sin*γ*cos(*β*−*α*). Substituting Eqs. (8)–(10) into Eq. (11), we obtain:

(12) $$\begin{array}{ll} E^{\sigma }\left(\rho ,\alpha ,z\right) & =C_{0}\int_{0}^{\gamma_{\max}}\int_{0}^{2\pi }{\text{e}}^{\text{i}\varphi_{0}}\sqrt{\cos\gamma }\left[Ae_{r}^{\prime }+Be_{\beta }^{\prime }\right]\\ & \;\times {\text{e}}^{\text{i}k\left(z\cos\gamma +\rho \sin\gamma \cos\left(\beta -\alpha \right)\right)}\sin\gamma d\beta d\gamma \end{array}$$

where $C_{0}=-\sqrt{2}ikf{\text{e}}^{\frac{\text{i}\pi }{4}}T_{xx}/8\pi$ , $\varphi_{0}=-2\pi \left(\sqrt{r^{2}+f^{2}}\right.\left.-f\right)/\lambda$ , $A={\text{e}}^{\text{i}\left(l_{p}+\sigma \right)\beta }+{\text{e}}^{-2\text{i}\left(\frac{\pi }{4}-\sigma \theta_{0}\right)}{\text{e}}^{\text{i}\left(l_{p}+l_{g}-\sigma \right)\beta }$ , and $B=i\sigma \left[{\text{e}}^{\text{i}\left(l_{p}+\sigma \right)\beta }-{\text{e}}^{-2\text{i}\left(\frac{\pi }{4}-\sigma \theta_{0}\right)}{\text{e}}^{\text{i}\left(l_{p}+l_{g}-\sigma \right)\beta }\right]$ . After straightforward mathematical operations, the field expressions in cylindrical coordinates can be derived as follows:

(13) $$\begin{array}{ll} E^{\sigma }\left(\rho ,\alpha ,z\right) & =\left[\begin{array}{c} E_{\rho }\\ E_{\alpha }\\ E_{Z} \end{array}\right]\\ & =C_{0}\int_{0}^{\gamma_{\max}}{\text{e}}^{\text{i}\varphi_{0}}{\text{e}}^{\text{i}k\left(z\cos\gamma +\rho \sin\gamma \cos\left(\beta -\alpha \right)\right)}\sqrt{\cos\gamma }\sin\gamma \\ & \;\times \int_{0}^{2\pi }\left[\begin{array}{c} A\text{cos}\gamma \cos\left(\beta -\alpha \right)-B\text{sin}\left(\beta -\alpha \right)\\ A\text{cos}\gamma \sin\left(\beta -\alpha \right)+B\text{cos}\left(\beta -\alpha \right)\\ A\sin\gamma \end{array}\right]\text{d}\beta d\gamma \end{array}$$

where *E*_*j*_ (*j* = *ρ*, *α*, *z*) denotes the component field along the *j*th coordinate axis. The integral over *β* in Eq. (13) can be calculated as follows:

(14) $$\begin{array}{ll} & \int_{0}^{2\pi }\cos\left(\beta -\alpha \right){\text{e}}^{\text{i}\left(l_{p}+\sigma \right)\beta }{\text{e}}^{\text{i}k\rho \sin\gamma \cos\left(\beta -\alpha \right)}\text{d}\beta \\ & \;=\pi i^{\left(l_{p}+\sigma +1\right)}{\text{e}}^{\text{i}\left(l_{p}+\sigma \right)\alpha }\left[J_{l_{p}+\sigma +1}\left(k\rho \sin\gamma \right)\right.\left.-J_{l_{p}+\sigma -1}\left(k\rho \sin\gamma \right)\right] \end{array}$$

(15) $$\begin{array}{ll} & \int_{0}^{2\pi }\sin\left(\beta -\alpha \right){\text{e}}^{\text{i}\left(l_{p}+\sigma \right)\beta }{\text{e}}^{\text{i}k\rho \sin\gamma \cos\left(\beta -\alpha \right)}\text{d}\beta \\ & \;=-\pi i^{\left(l_{p}+\sigma +2\right)}{\text{e}}^{\text{i}\left(l_{p}+\sigma \right)\alpha }\left[J_{l_{p}+\sigma +1}\left(k\rho \sin\gamma \right)\right.\\ & \;\left.+J_{l_{p}+\sigma -1}\left(k\rho \sin\gamma \right)\right] \end{array}$$

where *J*_*lp*+*σ*±1_ (*kρ*sin*γ*) is a Bessel function of the first kind with order *l*_*p*_ + *σ* ± 1.

Under RCP illumination (*σ* = −1), by substituting Eqs. (14) and (15) into Eq. (13) and based on the parameter condition *l*_*g*_ = −2*l*_*p*_, the radial, azimuthal, and longitudinal component fields *E*_*ρ*_, *E*_*α*_, and *E*_*z*_, respectively, can be expressed as:

(16) $$\begin{array}{ll} \left[\begin{array}{c} E_{\rho }\\ E_{\alpha }\\ E_{Z} \end{array}\right] & =2\pi i^{l_{p}}C_{0}\int_{0}^{\gamma_{\max}}\sqrt{\cos\gamma }\text{sin}\gamma {\text{e}}^{\text{i}\varphi_{0}}{\text{e}}^{\text{i}kz\text{cos}\gamma }{\text{e}}^{-\text{i}\left(\frac{\pi }{4}+\theta_{0}\right)}\left[\begin{array}{c} P_{\rho }\left(\alpha \right)\left[\left(\cos\gamma +1\right)J_{l_{p}}\left(k\rho \sin\gamma \right)-\left(\cos\gamma -1\right)J_{l_{p}-2}\left(k\rho \sin\gamma \right)\right]\\ P_{\alpha }\left(\alpha \right)\left[\left(\cos\gamma +1\right)J_{l_{p}}\left(k\rho \sin\gamma \right)+\left(\cos\gamma -1\right)J_{l_{p}-2}\left(k\rho \sin\gamma \right)\right]\\ P_{z}\left(\alpha \right)\sin\gamma J_{l_{p}-1}\left(k\rho \sin\gamma \right) \end{array}\right]\text{d}\gamma \end{array}$$

where the functions *P*_*j*_(*α*) for (*j* = *ρ*, *α*, *z*) are given by:

(17) $$P_{\rho }\left(\alpha \right)=\cos\left[\left(l_{p}-1\right)\alpha +\theta_{0}+\pi /4\right]$$

(18) $$P_{\alpha }\left(\alpha \right)=\sin\left[\left(l_{p}-1\right)\alpha +\theta_{0}+\pi /4\right]$$

(19) $$P_{z}\left(\alpha \right)=-2i\text{cos}\left[\left(l_{p}-1\right)\alpha +\theta_{0}+\pi /4\right]$$

The cylindrical wavefield ***E***_1_(*ρ*,*α*) = *E*_*ρ*_(*ρ*,*α*) ***e***_*ρ*_ + *E*_*α*_(*ρ*,*α*) ***e***_*α*_ given by Eq. (16), along with Eqs. (17) and (18), represents the transverse field on the focal plane *O*′*x*′*y*′, and can be written as:

(20) $$\begin{array}{ll} E_{1}\left(\rho ,\alpha \right) & =i^{l_{p}}C_{1}\psi_{1}\left(\gamma \right){\text{e}}^{-\text{i}\left(\frac{\pi }{4}+\theta_{0}\right)}\left({\text{e}}^{\text{i}\left(l_{p}\alpha +\frac{\pi }{4}+\theta_{0}\right)}\left|R\right\rangle \right.\\ & \;\left.+{\text{e}}^{-\text{i}\left(l_{p}\alpha +\frac{\pi }{4}+\theta_{0}\right)}\left|L\right\rangle \right) \end{array}$$

where

(21) $$\begin{array}{ll} \psi_{1}\left(\gamma \right) & =\int_{0}^{\gamma_{\max}}\sqrt{\cos\gamma }\sin\gamma {\text{e}}^{\text{i}\varphi_{0}}{\text{e}}^{\text{i}\mathrm{kz}\text{cos}\gamma }\\ & \;\times \left[\begin{array}{c} \left(\cos\gamma +1\right)J_{l_{p}}\left(k\rho \sin\gamma \right)-\left(\cos\gamma -1\right)J_{l_{p}-2}\left(k\rho \sin\gamma \right)\\ \left(\cos\gamma +1\right)J_{l_{p}}\left(k\rho \sin\gamma \right)+\left(\cos\gamma -1\right)J_{l_{p}-2}\left(k\rho \sin\gamma \right) \end{array}\right]\text{d}\gamma \end{array}$$

*C*_1_ = $\sqrt{2}$ *πC*_0_, $\left|L\right\rangle ={\text{e}}^{\text{i}\alpha }{\left[\begin{array}{cc} 1 & i \end{array}\right]}^{T}/\sqrt{2}$ , $\left|R\right\rangle ={\text{e}}^{-\text{i}\alpha }{\left[\begin{array}{cc} 1 & -i \end{array}\right]}^{T}/\sqrt{2}$ and represent RCP and LCP in the cylindrical coordinate system, respectively, and the functions *P*_*ρ*_(*α*) and *P*_*α*_(*α*) are substituted with |*L*⟩ and |*R*⟩. According to Eq. (20), ***E***_1_(*ρ*,*α*) represents the equally weighted superposition of two orthogonal CPs e^−*i* *lpα*^|*L*⟩ and e^*i* *lpα*^|*R*⟩ with the initial phases −(*π*/4 + *θ*_0_) and (*π*/4 + *θ*_0_), respectively. This indicates that ***E***_1_(*ρ*,*α*) forms a linearly polarized VB of order *l*_*p*_, with the polarization state controlled by the orientation angle *θ*_0_ of the meta-atoms. When *l*_*p*_ = 1 and *θ*_0_ = −*π*/4, the VV modes e^−*i* *α*^|*L*⟩ and e^*i* *α*^|*R*⟩, respectively, can be achieved with their corresponding vanishing phase differences. Therefore, the superposition of the two VV modes forms radially polarized VBs. In this case, according to Eq. (19), the function *P*_*z*_(*α*) is equal to −2*i*, indicating the presence of a longitudinal component near the focus, leading to a tighter spot beyond the diffraction limit. When *θ*_0_ = *π*/4, the phase difference between the two VV modes is *π*. Consequently, their superposition creates azimuthally polarized VBs on the focal plane. Simultaneously, *P*_*z*_(*α*) = 0, indicating the absence of any longitudinal component near the focus. Similarly, when *θ*_0_ is equal to 0 and *π*/2, slanted polarization VBs of 45° and 135° can be obtained on the focal plane, respectively. In addition, focused VBs of *π*-radial, *π*-45°, *π*-azimuthal, and *π*-135° can be generated when *l*_*p*_ = −1 and *θ*_0_ = −*π*/4, 0, *π*/4, and *π*/2, respectively.

Under LCP illumination, by using the same mathematical calculation as that for deriving Eq. (20) but the parameter condition *l*_*g*_ = 2*l*_*p*_ for *σ* = 1, the corresponding cylindrical wavefield ***E***_2_(*ρ*,*α*) on the focal plane can be expressed as:

(22) $$\begin{array}{ll} E_{2}\left(\rho ,\alpha \right) & =i^{l_{p}}C_{1}{\text{e}}^{-\text{i}\left(\frac{\pi }{4}-\theta_{0}\right)}\left(\psi_{2}\left(\gamma \right){\text{e}}^{\text{i}\left(l_{p}\alpha +\frac{\pi }{4}-\theta_{0}\right)}\left|L\right\rangle \right.\\ & \;\left.+\psi_{3}\left(\gamma \right){\text{e}}^{\text{i}\left(3l_{p}\alpha -\frac{\pi }{4}+\theta_{0}\right)}\left|R\right\rangle \right) \end{array}$$

where

(23) $$\begin{array}{ll} \psi_{2}\left(\gamma \right) & =\int_{0}^{\gamma_{\max}}\sqrt{\cos\gamma }\sin\gamma {\text{e}}^{\text{i}\varphi_{0}}{\text{e}}^{\text{i}\mathrm{kz}\text{cos}\gamma }\\ & \;\times \left[\begin{array}{c} \left(1-\cos\gamma \right)J_{l_{p}+2}\left(k\rho \sin\gamma \right)+\left(\cos\gamma +1\right)J_{l_{p}}\left(k\rho \sin\gamma \right)\\ \left(\cos\gamma -1\right)J_{l_{p}+2}\left(k\rho \sin\gamma \right)+\left(\cos\gamma +1\right)J_{l_{p}}\left(k\rho \sin\gamma \right) \end{array}\right]\text{d}\gamma \end{array}$$

(24) $$\begin{array}{ll} \psi_{3}\left(\gamma \right) & =\int_{0}^{\gamma_{\max}}\sqrt{\cos\gamma }\sin\gamma {\text{e}}^{\text{i}\varphi_{0}}{\text{e}}^{\text{i}kz\text{cos}\gamma }\\ & \;\times \left[\begin{array}{c} \left(1-\cos\gamma \right)J_{3l_{p}-2}\left(k\rho \sin\gamma \right)+\left(\cos\gamma +1\right)J_{3l_{p}}\left(k\rho \sin\gamma \right)\\ \left(\cos\gamma -1\right)J_{3l_{p}-2}\left(k\rho \sin\gamma \right)+\left(\cos\gamma +1\right)J_{3l_{p}}\left(k\rho \sin\gamma \right) \end{array}\right]\text{d}\gamma \end{array}$$

***E***_2_(*ρ*,*α*) includes two orthogonal CP vortex beams with topological charges of unequal absolute values. The superposition of the two VV modes results in the generation of hybrid-order VBs. The initial phases of the two CP light are (*θ*_0_−*π*/4) and −(*θ*_0_−*π*/4), and the polarization states of the generated hybrid-order VBs are determined by the initial orientation angle *θ*_0_ of the meta-atom. Therefore, the proposed metasurface design provides a novel method for generating diverse and focused structured light fields.

## 3 Numerical simulation

To validate the proposed design, we perform FDTD simulations involving 2D parameter sweeps over the transmitted light field of a meta-atom, that is, an a-Si:H rectangular nanopillar. The height and lattice period of the meta-atoms were *H* = 480 nm and *P* = 380 nm, respectively, and the side length of the nanopillar varied from 80 nm to 330 nm in steps of 1 nm. The refractive index and extinction coefficient of the nanopillars were *n* = 3.744 and *κ* = 0.000 at a wavelength of 800 nm, respectively. The periodic nanopillar array was illuminated with *x-* and *y*-linearly polarized light, and the propagation phases *φ*_*xx*_(*x*,*y*) and *φ*_*yy*_(*x*,*y*) were obtained as functions of the length and width of the nanopillars. From the simulation results, we selected eight QWP meta-atoms, represented by the small orange rectangles at the bottom of Figure 1(c). The propagation phases *φ*_*xx*_(*x*,*y*) of these eight meta-atoms ranged from 0 to 2*π* with an incremental step of 0.25*π.* The phase retardation |*φ*_*yy*_−*φ*_*xx*_| between two LP excitations approached 0.5*π*. The transmission coefficient of each meta-atom was greater than 83 %, and the transmission ratio *T*_*xx*_/*T*_*yy*_ was approximately 1. Table 1 lists the dimensions of the eight selected meta-atoms, corresponding propagation phases *φ*_*xx*_(*x*,*y*), and phase retardation |*φ*_*yy*_−*φ*_*xx*_|.

**Table 1:** Dimensions of eight selected meta-atoms and their corresponding phases.

Specifications	*L*(nm)	*W*(nm)	*φ*_*xx*_(*π*)	|*φ*_*yy*_−*φ*_*xx*_|(*π*)
1	140	170	−0.01	0.51
2	95	275	0.24	0.49
3	145	250	0.49	0.49
4	275	95	0.73	0.49
5	155	130	1.03	0.50
6	160	135	1.22	0.53
7	170	140	1.48	0.51
8	135	160	1.75	0.53

Using these meta-atoms, we designed four metasurface samples labeled S_A_, S_B_, S_C_, and S_D_ to generate the focused radial, azimuthal, *π*-radial, and *π*-azimuthal VBs, respectively. The vortex topological charge *l*_*p*_ of samples S_A_ and S_C_ was set as +1 and −1, respectively, with both samples sharing the same initial orientation angles, that is *θ*_0_ = −*π*/4. Counterclockwise rotation of the initial angle *θ*_0_ of meta-atoms in sample S_A_ by 90° yielded samples S_B_ for azimuthal VBs. Similarly, rotation of the meta-atoms of sample S_C_ by *θ*_0_ = 90° yielded sample S_D_ for *π*-azimuthal VBs. Each sample consisted of 100 equally spaced concentric rings, in which the meta-atoms were evenly arranged. The diameter of each metasurface sample was 76 μm, and the focal length *f* was set as 150 μm. In the FDTD simulations, we first calculated the near-field light distribution for each sample under RCP illumination and then projected it onto the far-field plane to obtain the focused VBs of Bell-like states.

**Figure 2:**
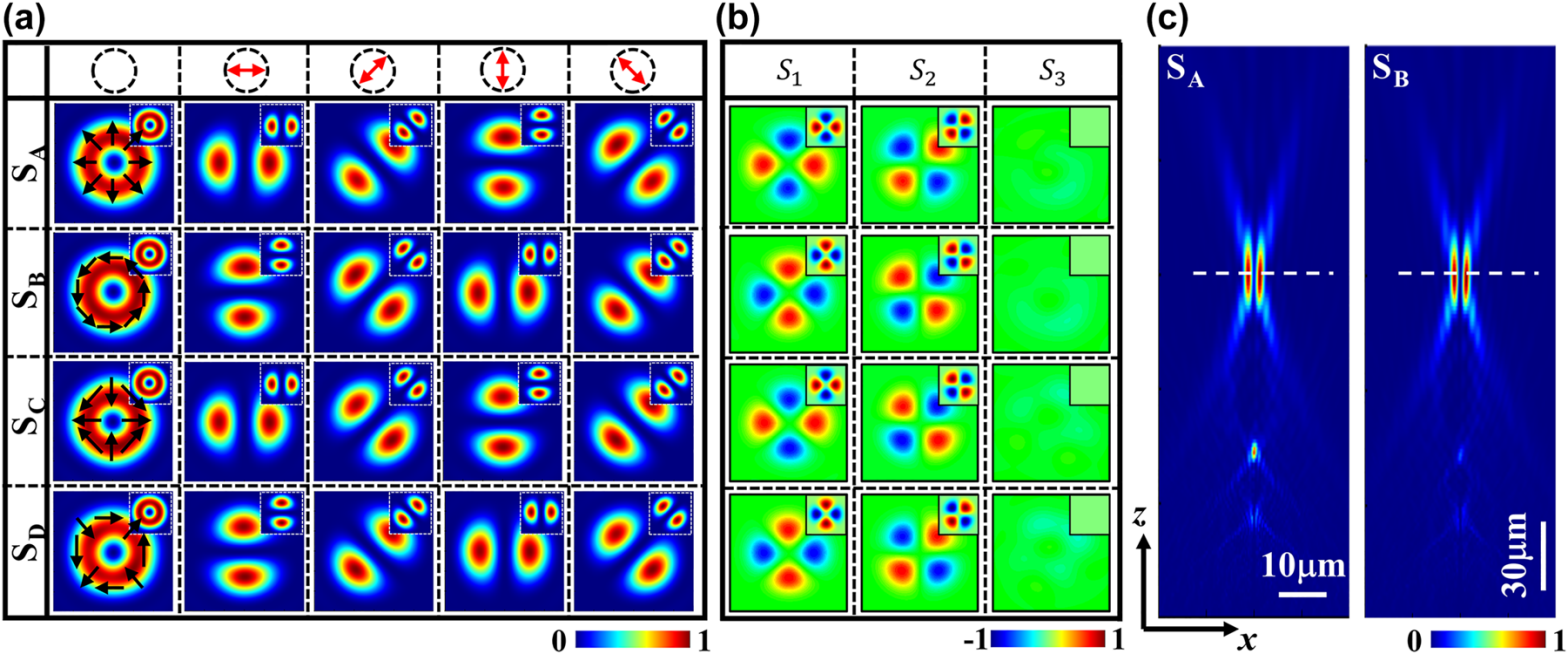
Simulated results for focused VBs generated by the metasurfaces. (a) Intensity images of VBs of Bell-like states generated under RCP illumination. The top to bottom rows show the intensities of Bell-like states |TM⟩_1_, |TE⟩_1_, |HE^e^⟩_1_, and |HE^o^⟩_1_, respectively, generated by samples S_A_, S_B_, S_C_, and S_D_. The left to right columns show the total intensity and *x*-, 45°, *y*-, and 135° component intensities. (b) The top to bottom patterns show the Stokes parameters of the Bell-like states |TM⟩_1_, |TE⟩_1_, |HE^e^⟩_1_, and |HE^o^⟩_1_. (c) The *x*–*z* cross-section images of the total intensity of VBs generated by sample S_A_ and sample S_B,_ respectively.

Figure 2(a) shows the simulated fields of the four metasurfaces under RCP incidence, with the corresponding theoretical results obtained using MATLAB programming shown in the insets in the upper-right corner. The top row of the figure shows the transmission direction of the polarizer. The top to bottom rows show the intensities of the Bell-like states generated by the four samples |TM⟩_1_, |TE⟩_1_, |HE^e^⟩_1_, and |HE^o^⟩_1_. The left to right columns show the total intensity and component intensities of the *x*-, 45°, *y*-, and 135° polarizations, respectively, with the polarization states overlaid on the doughnuts of the total intensities. The observed patterns reveal that the component intensities consist of two lobes with various orientations separated by dark lines, whereas the vortex patterns of the total intensities exhibit central dark cores owing to the polarization singularity. Moreover, the distribution directions of the two lobes in the component fields of samples S_A_ and S_B_ are perpendicular, indicating that the polarization states of the corresponding VBs are orthogonal. This correlation also holds for the focused VBs generated by samples S_C_ and S_D_, consistent with the theoretical conclusion that the polarization states of the VBs are controlled by the initial orientation angle *θ*_0_ of the meta-atoms.

To characterize the polarization behavior of the generated VBs, we calculated their Stokes parameters (*S*_0_, *S*_1_, *S*_2_, and *S*_3_), where *S*_1_ = *I*_*H*_−*I*_*V*_, *S*_2_ = *I*_*D*_−*I*_*A*_, and *S*_3_ = *I*_*L*_−*I*_*R*_. Here, *I*_*H*_, *I*_*D*_, *I*_*V*_, and *I*_*A*_ represent the intensities of the *x*-, 45°, *y*-, and 135° components, respectively; and *I*_*L*_ and *I*_*R*_ denote the intensities of the LCP and RCP components. Figure 2(b) shows the Stokes parameters simulated using FDTD. The patterns of *S*_1_ and *S*_2_ consist of four lobes, and *S*_3_ is approximately 0, indicating that the generated VBs are equally weighted superpositions of two CP beams, and the eight selected meta-atoms appear to be nearly ideal QWP. The corresponding results calculated through MATLAB programming are shown in the upper-right insets, consistent with the FDTD simulation results. In Figure 2(c), the left and right patterns show the *x*–*z* cross-section images of the total intensity of VBs generated by sample S_A_ and sample S_B_, respectively, in which the white dashed lines denote the positions of the focal plane.

## 4 Experimental measurement

**Figure 3:**
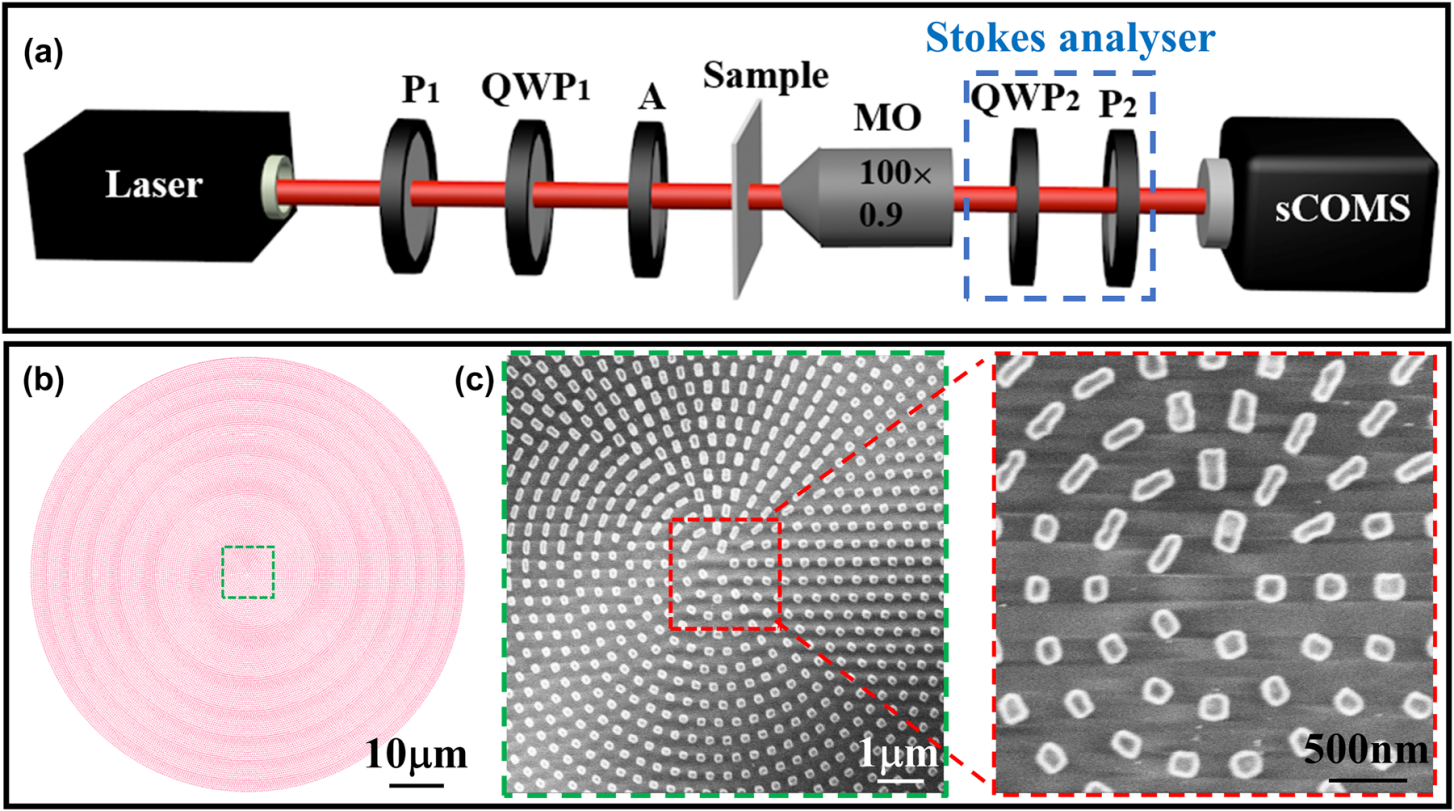
Diagram of the optical setup and SEM image of the metasurface. (a) Schematic of the experimental setup. QWP, quarter-wave plate; A, attenuator; MO, microscope objective; P, linear polarizer. (b) Schematic structure of sample S_A_ derived from FDTD software. (c) SEM image of sample S_A_ and enlarged view.

Figure 3(a) illustrates the experimental setup for measuring the focused VBs generated by the metasurface samples. The light source emitted a linearly polarized light beam with a wavelength *λ* of 800 nm, which was calibrated using a polarizer (*P*_1_) and converted to CP light using a quarter-wave plate (QWP_1_). The beam intensity was modulated using an attenuator A, and the samples were mounted on a three-dimensional piezo nanometer stage (PI E727). A microscopic objective lens (MO) was used to collect and image the VB fields, and the intensity patterns of the focused VBs on the focal plane were recorded using an s-CMOS detector (Zyla-5.5) placed on the image plane. Each designed sample was optimized through an FDTD simulation of the generated VBs, and the sample patterns with nanopillar arrays were exported for fabrication using electron beam lithography. Figure 3(b) schematically illustrates the structure of sample S_A_, and Figure 3(c) shows the scanning electron microscopy (SEM) images of the sample (local) along with an enlarged view. To experimentally measure the Stokes parameters, another quarter-wave plate (QWP_2_) and polarizer (*P*_2_) were placed in front of the s-CMOS to form a Stokes analyzer. The component intensity patterns of the *x*-, 45°, *y*-, and 135° polarizations were obtained by removing QWP_2_ and rotating the transmission direction of *P*_2_. Subsequently, the Stokes parameters *S*_1_ and *S*_2_ were calculated. The component intensities of the RCP and LCP were acquired by adjusting the angle between the fast axis of QWP_2_ and transmission direction of *P*_2_, and the Stokes parameter *S*_3_ was thus obtained.

**Figure 4:**
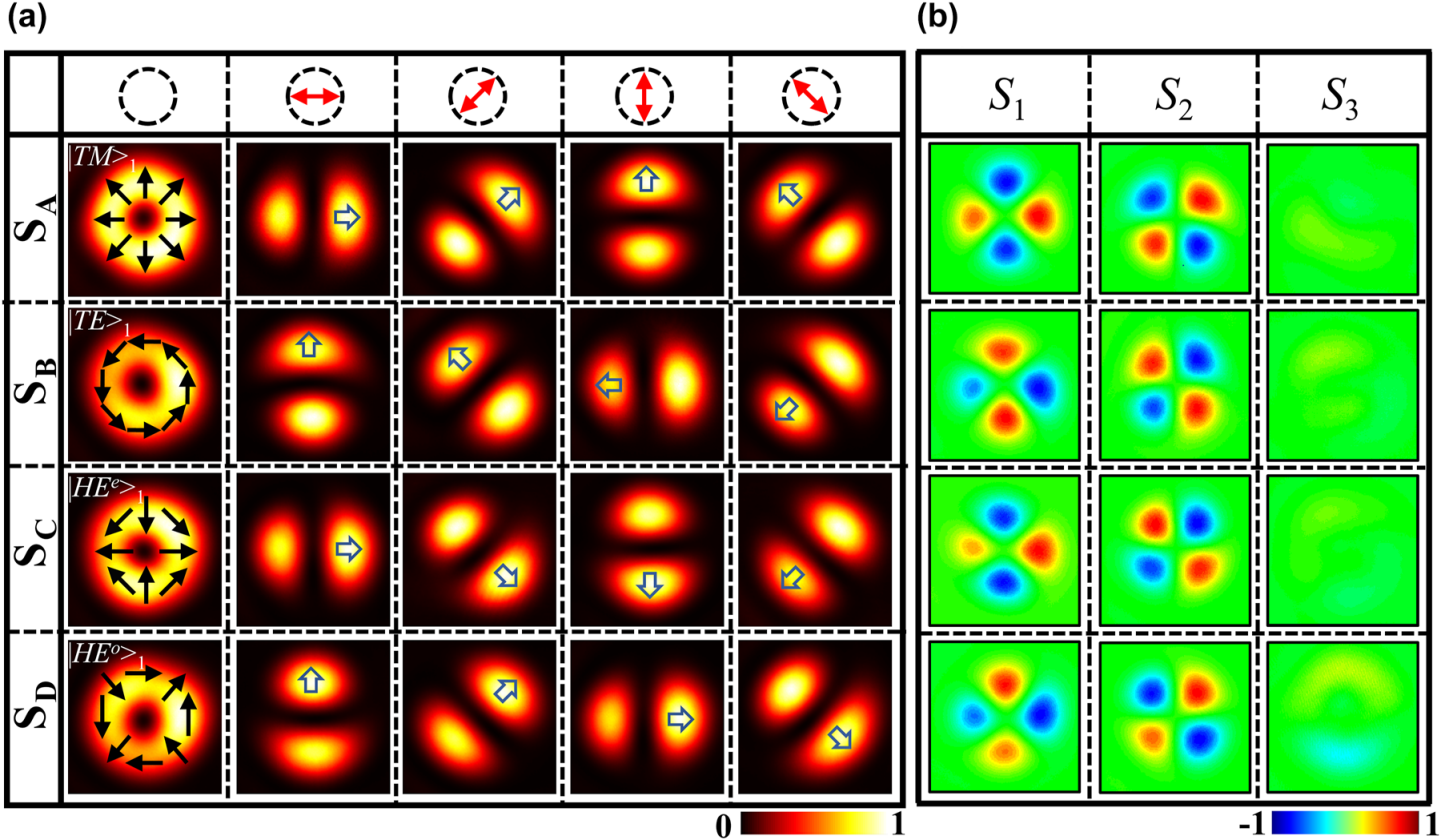
Experimental results for focused VBs generated by the metasurfaces. (a) Intensity images of VBs of Bell-like states generated by four samples under RCP incidence. The intensity patterns from top to bottom rows denote the radially, azimuthally, *π*-radially, and *π*-azimuthally polarized VBs. The left to right columns show the total field and intensity patterns of *x*-, 45°, *y*-, and 135° polarizations. (b) Stokes parameters of generated VBs of Bell-like states.

Figure 4(a) shows the experimental intensities of the focused Bell-like states generated by the four samples under RCP illumination. The top row indicates the transmission direction of *P*_2_. The patterns from top to bottom rows represent the intensities |TM⟩_1_, |TE⟩_1_, |HE^e^⟩_1_, and |HE^o^⟩_1_. From left to right, the patterns depict the total intensity with a doughnut distribution overlaid by the polarization states and component intensities. Each component intensity pattern exhibits two lobes with high symmetry and uniform intensity distribution, and the orientations of the two lobes (denoted by hollow arrows) rotate in the transmission direction of polarizer *P*_2_, highlighting the nonseparability of the polarization and spatial modes of the generated VBs. The orientations of lobes in the component intensities |TM⟩_1_ and |TE⟩_1_ are parallel and perpendicular to the transmission direction of *P*_2_, respectively, although they rotate with the rotation of the transmission direction of *P*_2_. In contrast, the orientations of the two lobes in |HE^e^⟩_1_ and |HE^o^⟩_1_ rotate in the direction opposite to the rotation of *P*_2_. The Stokes parameters *S*_1_, *S*_2_, and *S*_3_ were measured experimentally, as shown in Figure 4(b). The experimental results of the intensity patterns of the VBs of Bell-like states and Stokes parameters agree with those of the FDTD simulation, demonstrating that the proposed metasurface design can generate high-quality focused VBs of Bell-like states under RCP illumination.

## 5 Discussion and conclusion

The comprehensive analyses described above demonstrated that the proposed QWP metasurfaces can generate focused VBs of Bell-like states under RCP illumination. To explore the extended functionalities of the metasurfaces, we explored the spatially structured fields generated by the samples under CP illumination with reversed chirality. When subjected to LCP with *σ* = 1, the propagation phase profile *φ*_*p*_(*x*,*y*) remained unchanged, whereas the PB phase *φ*_*p*_(*x*,*y*) was inverted because of its dependence on the chirality of the CP light. According to the parameter condition *l*_*g*_ = 2*l*_*p*_, the topological charges of the co- and cross-polarized components exhibited unequal absolute values, and the corresponding superposition of the two VV modes produced hybrid-order VBs. Specifically, for sample S_A_, the orbital angular momentum (OAM) states of the co- and cross-polarized components were |*l*_*p*_⟩ = |+1⟩ and |*l*_*p*_ + *l*_*g*_⟩ = |+3⟩, respectively, and the superposition of the two orthogonal VV modes |*L*, +1⟩ and |*R*, +3⟩ generated focused hybrid-order VBs. Similarly, for sample Sc, the superposition of the two corresponding orthogonal VV modes, |*L*, −1⟩ and |*R*, −3⟩, produced a hybrid-order VB. Moreover, the polarization states of the generated hybrid-order VBs were controlled by the initial orientation angle *θ*_0_ of the meta-atom.

**Figure 5:**
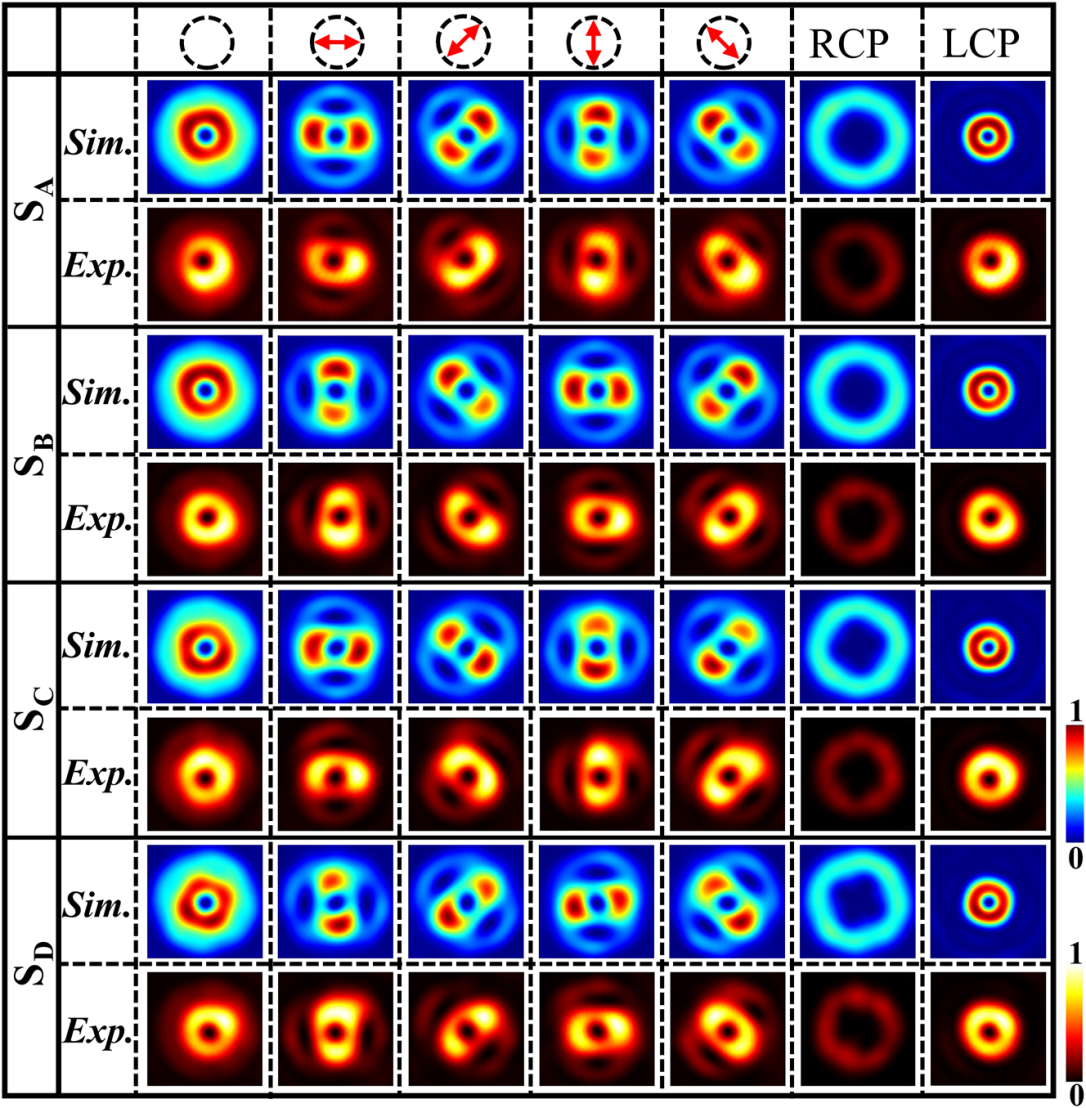
Simulation and experimental results obtained using four samples under LCP illumination. From left to right, the patterns show the total field and intensities of the *x*-, 45°, *y*-, 135°, RCP, and LCP components.

Figure 5 shows the simulation and experimental results for the four samples under LCP illumination. Columns from left to right show the total intensity and intensities of the *x*-, 45°, *y*-, 135°, RCP, and LCP components. The top row in the figure shows the transmission direction of *P*_2_. The patterns from the top to bottom show the focused hybrid-order VBs generated by the four samples. The total intensity was superposed by a pair of vortex beams with different radii, given the unequal topological charges of the two VV modes. Interestingly, the characteristics of *x*-, 45°, *y*-, and 135° component intensities lay in unique lobe patterns that were not completely divided by the dark line, differing from those of the VBs of Bell-like states. The RCP and LCP components exhibited doughnut-shaped intensity distributions. Additionally, the two unique lobes rotated in the transmission direction of *P*_2_, similar to the lobes of the VBs of the Bell-like states. Overall, the four samples could generate hybrid-order VBs corresponding to the superposed states of different OAMs under LCP illumination.

Comparing the intensity patterns in Figures 2(a), 4(a), and 5, we may find that the simulated and experimental light distributions appear to be somehow nonuniform, exhibiting some differences from the theoretical calculations. Different factors may lead to the differences. In the FDTD simulations, the phase retardations of the eight selected meta-atoms slightly deviated from the perfect QWP, and their propagation phases deviated from the linear distribution of the ideal phase increment of 0.25*π*. These deviations may be a probable reason for the discrepancy between the simulated and experimental results and the theoretical calculations. In the principle of the general metasurface design, the eight QWP meta-atoms are selected from the simulation results performed under rigorously periodic boundary conditions. However, such rigorous conditions are not ensured owing to the size variations of the eight selected meta-atoms constituting the metasurface; this may inevitably lead to the cross-talk between the meta-atoms and the uncontrollable residual waves in the transmitted light fields [66], deteriorating the simulated and experimental results. Besides, the fabrication errors of the meta-atoms such as the rough surface, rugged edge and dimension deviation may induce even more discrepancy between the experimental and theoretical results. In addition, the imperfect performance of the optical components as well as inevitable alignment and rotation errors may also lead to the nonuniform distribution of light field.

In addition, the efficiencies of the proposed metasurfaces have been performed by the FDTD simulation. The calculations were based on the efficiency defined as the ratio of the integral of transmitting energy flow over the monitored area to the incident power on the illuminating area. In the simulation, the diameter of the samples was 76 μm, and the illumination area of the incident light was 77 μm × 77 μm. The monitored area on the far-field focal plane is 12 μm × 12 μm, which is about 2.5 times of the doughnut diameter (about 4.8 μm) of the focused VBs. Specifically, the efficiencies of the radial and azimuthal polarized VBs produced by sample S_A_ and S_B_ are 40.873 % and 38.015 %, respectively.

It is worth noting that the PB phases with equal and opposite signs cause the conjugated symmetry of the transmitted wavefields under CP illumination, resulting in the spin-locking for LCP and RCP beams. The functionalities of PB metasurfaces with meta-atoms of identical dimensions are often locked and mirrored for the two CP beams owing to the spin-locking. To extend the functionalities, the spin-decoupled metasurfaces have been developed, and they are generally categorized into two types [44]. One type is designed by utilizing the merged meta-structures, which merges or interleaves several different PB metasurfaces in a compact configuration, to achieve powerful functionalities. Based on this approach, various spin-decoupled metasurfaces have been proposed to achieve multiple functionalities [55]. Although the integration of different functionalities can be achieved with this type of spin-decoupled metasurface, the spin-locked limitations of the PB metasurfaces still exit, which may bring about the issues of low operating efficiencies and functionality cross-talking [44]. The other type of spin-decoupled metasurfaces is designed by combining the propagation and PB phases with high efficiency and low-crosstalk, and simultaneously, the spin-locking between two orthogonal CP components is also decoupled. In the design of this HWP metasurface, LP light is used as the illumination, and the transmitted light field contains only the cross-polarized components, but no co-polarized components. By adding the spin-independent propagation phase to the PB phases of the conjugate symmetry, the spin-locking is broken and the two orthogonal cross-polarized components are decoupled. Based on this approach, various multi-functional spin-decoupled HWP metasurfaces have been proposed and have enabled applications in a wide range of frontier fields [67], [68], [69]. Specifically, when the spin-decoupled HWP metasurfaces are used to independently manipulate the two orthogonal VV modes, their superposition will generate VBs such as perfect vector beams [32], [70], terahertz vector beams [71], and vectorial optical fields [72]. It is noticed that for such spin-decoupled HWP metasurface, the topological charge *l*_*p*_ of the propagation phase must not be equal to zero, and the propagation and PB phases are both used to construct helical phase profiles. Then, the two orthogonal VV modes with different topological charges were formed, and their superposition generates the hybrid-order Poincaré beams. Therefore, the spin-decoupled HWP metasurfaces combining the propagation and PB phases cannot be used to generate the HOP beams, including the VBs of Bell-like states. For the HOP beams to be generated, the HWP PB metasurfaces [66], [73] or the merging HWP metasurfaces have to be used [15], [74].

In summary, the proposed novel dielectric QWP-metasurface with multifunctional capabilities could successfully generate a complete set of focused VBs of Bell-like states under RCP illumination. By leveraging the propagation phase imparted to the co-polarized component, hyperbolic and helical phase profiles were constructed, whereas the PB phase acting on the cross-polarized component was used to yield another helical phase profile. Thus, simultaneous manipulation of the co- and cross-polarized components enabled the generation of the desired focused VBs. The proposed QWP-metasurface, designed by combining propagation and PB phases, could simultaneously manipulate the two VV modes to generate focused VBs of Bell-like states under RCP illumination, differing from the previous QWP-metasurface. VBs of linear polarizations at the equator of the HOP sphere were obtained by manipulating the initial orientation angle *θ*_0_ of the meta-atoms. In addition, focused hybrid-order VBs of the superimposed states of different OAMs were generated under LCP illumination. The consistency between the simulation and experimental results demonstrates the feasibility of the proposed schemes. The findings of this study have practical implications for the miniaturization and integration of related optical systems, with potential applications in classical physics and quantum science.
